# Factors influencing frailty in older adults admitted to a tertiary level hospital of Nepal

**DOI:** 10.1002/alz.087869

**Published:** 2025-01-09

**Authors:** Prabha Shrestha, Sarina Shakya, Radha Acharya

**Affiliations:** ^1^ Global Brain Health Institute, San Francisco, CA USA; ^2^ Kathmandu University School of Medical Sciences, Dhulikhel Nepal; ^3^ Kathmandu University School of Medical Sciences, Dhulikhel, Bagmati Nepal; ^4^ Kathmandu University School of Medical Sciences, DHULIKHEL, Bagmati Nepal

## Abstract

**Background:**

Frailty is significantly associated with incidence of Alzheimer’s disease and dementia and death ^(1)^. The worldwide incidence of frailty and pre frailty was estimated as 43.4 and 150.6 new cases per 1000 person‐years, respectively^(2)^. Risk of frailty is high in Low and Middle Income countries ^(3)^ 65%‐71% of the community dwelling older adults had reported frailty in Nepal^(4)^. We aimed to find out the frequency of frailty and it’s associated factors in older adults admitted to a tertiary level hospital of Nepal.

**Method:**

We conducted a cross sectional analytical study with the estimated sample size of 109. Ethical approval was obtained from ethical review committee Kathmandu University School of Medical Sciences. Data were collected from the older adults admitted to general wards via face to face interview after obtaining informed consent. Frailty was assessed by Groningen Frailty Index (GFI) which has total score of 15 and the participants scoring four or more were considered frail (4). Binary logistic regression analysis was done to examine the association between independent variables and frailty.

**Result:**

Total 109 older adults participated in the study. The mean age was 72.5 (60‐90) years. Half of the participants (51.4%) were male. Nearly two third (60.6%) among them met the criteria of frailty. Mean score of frailty was 4.9 with SD 3.55. On the Binary logistic analysis, the model predicted frailty with 38% of variance (Nagelker R Square = 0.38) in the outcome. Female were at risk of frailty with odd ratio of 3.48 (p = .014) history of fall within a year predicted frailty with odd ratio 4.24 (p = .043) and the people who perceived their health as good reduced the risk of frailty with odd ratio 0.34 (p = 0.10). However, age, presence of chronic illness and number of daily drug consumption could not predict frailty in this population.

**Conclusion:**

These result raise red flags for the incidence of dementia in Nepal, as frailty is a notable precursor to worsening cognitive status. Timely management of frailty in Nepal is imperative. Further research is needed to identify the best ways to address frailty in this setting.

